# Integrating molecular and structural findings: Wnt as a possible actor in shaping cognitive impairment in Cornelia de Lange syndrome

**DOI:** 10.1186/s13023-017-0723-0

**Published:** 2017-11-21

**Authors:** Laura Avagliano, Paolo Grazioli, Milena Mariani, Gaetano P. Bulfamante, Angelo Selicorni, Valentina Massa

**Affiliations:** 1 Department of Health Sciences, San Paolo Hospital Medical School University of Milan, Via A. di Rudinì, 8, 20142 Milan, Italy; 2 UOC Pediatria, ASST Lariana, Como, Italy

**Keywords:** Brain abnormalities, Cornelia de Lange syndrome, Wnt pathway

## Abstract

Cornelia de Lange Syndrome (CdLS) is a choesinopathy: a severe genetic disorder caused by mutations in the cohesin complex genes. The phenotype is characterized by typical facial dysmorphism, growth impairment and multiorgan abnormalities including brain alterations. Wnt pathway is known to play a fundamental role in central nervous system development and it has been shown that Wnt pathway is disrupted in CdLS animal models and patients cells. In this review we investigate the possible link between Wnt pathway disruption and brain abnormalities in Cornelia de Lange Syndrome as such molecular impairment could lead to an abnormal embryonic development resulting in brain abnormalities (i.e. microcephaly, cerebellar hypoplasia, abnormal cortical development) in patients with Cornelia de Lange Syndrome.

## Background

CdLS is a choesinopathy disorder caused by heterozygous or X-linked mutations in the cohesion core subunits *SMC1A*, *SMC3*, *RAD21*, or in cohesion components *NIPBL* and *HDAC8* [1–6]. CdLS manifests with typical facial dysmorphism (hirsute forehead, arched eyebrows with synophyrs, short nose with depressed nasal bridge, anteverted nares, long and smooth philtrum, thin lips, downwards turning corners of the mouth), growth impairment and multiorgan abnormalities including limb anomalies, congenital heart defects, gastrointestinal disease and brain alterations. Developmental disability invariably affects patients with CdLS even if about 20-30% of patients show mild impairment. Even if clinical signs of the neurodevelopmental impairment of multifactorial origin might be related to anatomical brain abnormalities, only few studies report brain features.

Wnts are secreted glycoproteins that activate signaling cascades involved in cell fate specification, polarity and migration, implicated in many aspects of embryo development. Wnt genes and signaling proteins are known to play a major role in fetal brain development [7, 8] and Wnt signaling pathway alterations have been associated to a number of central nervous system diseases [7].

The present review focuses on brain findings of patients affected by CdLS, exploring a possible correlation with Wnt signaling.

## Brain development and Wnt pathway

The human central nervous system (brain and spinal cord) is formed during the process known as neurulation that occurs between 20 and 27 days post-fertilization [9]. In the previous developmental phase, called gastrulation, the ectoderm is formed, which will thicken in response to an array of molecular signals released by the underlying notochord, originating the neural plate. This plate of ectodermal cells will form the neural tube by elevating, juxtaposing and fusing along the midline, thanks to a process of folding up on its anterior–posterior axis. At the end of the fusion along the midline, a hollow tube forms, called the neural tube [10].

During gastrulation, as the neural plate is forming, neural crest cells are organized at the edge of the prospective neural plate [11]. Neural crest cells are initially located within the dorsal part of the neural tube, at the edge of the neural plate, bordering between neuronal and non-neuronal ectoderm; during neural tube closure, neural crest cells delaminate from the dorsal neural tube along the embryo body axis and migrate, differentiating into multiple cell types, such as neurons and glial cells of the peripheral nervous system and structures such as cranial bones, cranial cartilage, dentin and dental pulp [12, 13].

Just before neural tube closure, the anterior extremity of the tube begins to expand forming the three primary brain vesicles [9]. The prosencephalon is the most anterior of these vesicles, which will become the forebrain. The middle vesicle is called the mesencephalon and represents the precursor of midbrain structures. The rhombencephalon is the most posterior vesicle and represents the embryonic hindbrain [9]. These three primary vesicles are visible approximately at day 28 post-fertilization. The prosencephalon and rhomboencephalon soon divide forming the secondary brain vesicles: telencephalon and diencephalon rostrally and metencephalon and myelencephalon caudally. All these structures are visible by day 49 post-fertilization [9]. Cerebral hemispheres develop because of sagittal folding and division of the telencephalon and rapidly expand and completely cover the diencephalon; the telencephalon augments in dimension for the active formation and differentiation of neurons and glia: from the germinal neuroepithelium, stem cells give rise to neurons and non-neuronal cells, thereafter immature neurons migrate to colonize the forebrain, midbrain, hindbrain in different spatial distribution of cortical layers, nuclei, and ganglia. At nine weeks of gestation, brain lobes are formed, and then sulci and gyri develop, together with the formation of the corpus callosum, representing the inter-commissural connections [9]. The cortex and medullary center develop, as well as the basal ganglia, lamina terminalis, hippocampus, corpus striatum and olfactory system.

The diencephalon will form the thalamus, epithalamus, hypothalamus, subthalamus, neurohypophysis, pineal gland, retina, optic nerve and mamillary bodies.

The mesencephalon develops into the lamina quadrigemina, the cerebral peduncles and the cerebral aqueduct. Pons and cerebellum originate from the metencephalon whereas the medulla oblongata develops from the myelencephalon.

To warrant a proper formation and function of the central nervous system, the complex developmental process described above must be coordinated by the developmental activity of a vast number of genes and proteins. The Wnt pathway has been shown to intervene in all steps of brain developmental process [8]: for example Wnt signals are involved in generation and migration of neural crest cells [12–15] implicated in the cranio-facial development [16–19]; Wnt proteins provide positional information within the embryo for anterior-posterior axis specification of the neural plate [20], regulating morphogenesis of the neural tube [21–23]: anterior-posterior specification of the neural plate and neural tube is dependent on graded Wnt signaling [8] requiring an inhibition of Wnt signaling in the anterior zone for proper anterior-posterior patterning of the early central nervous system development [8]; Wnt signals also intervene in neuronal cells development participating in neuron formation from the neuronal stem cells, neuronal proliferation and specification, neuronal migration and maturation including axon growth, dendrites formation and synaptogenesis [7, 8].

**Fig. 1:**
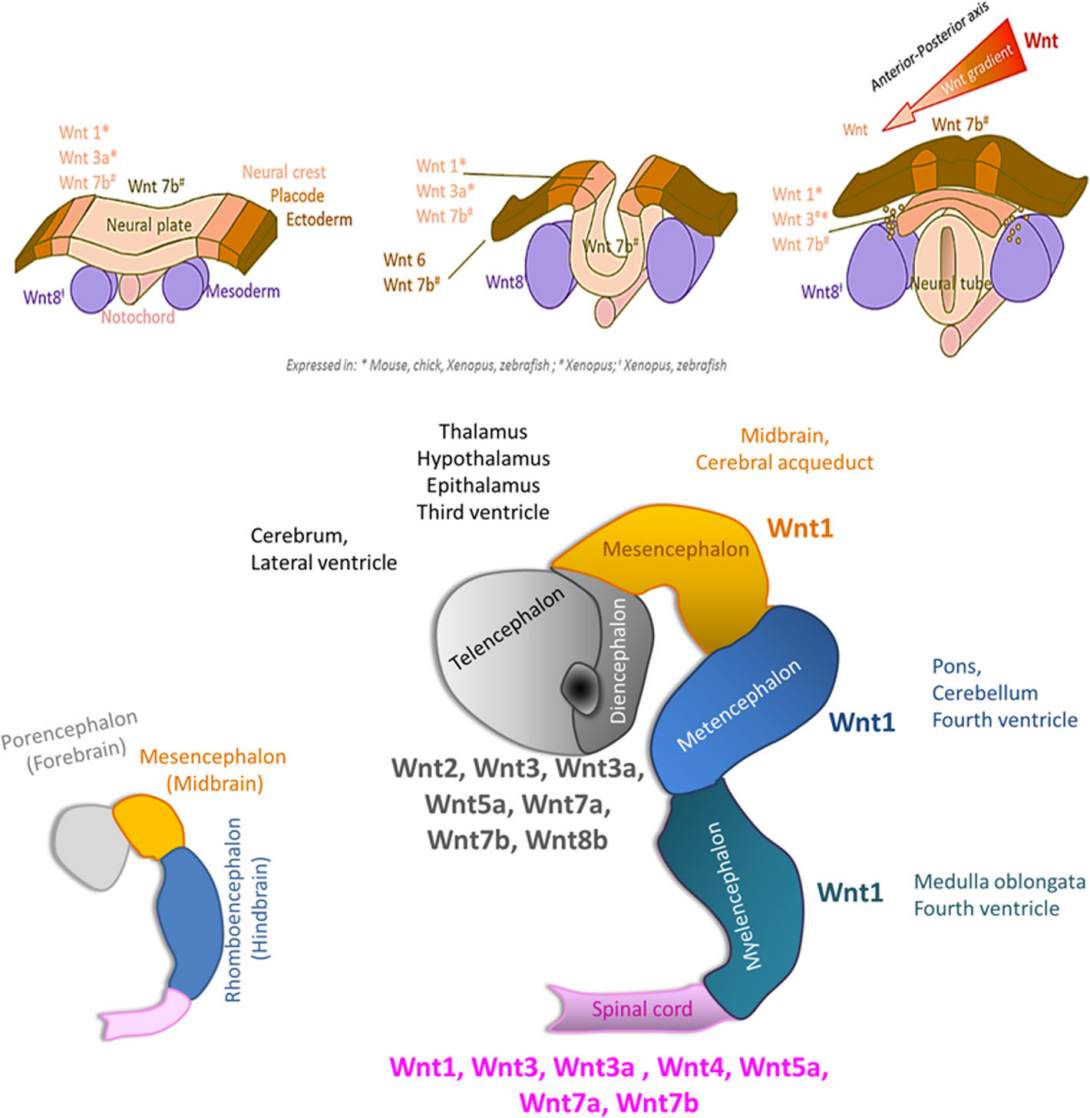
Wnt genes expression during the brain developmental process, from gastrulation to the differentiation of the structures forming the central nervous system. Note the spatial and temporal overlapping of the gene expression pattern

Expression of the Wnt genes during brain development is summarized in Fig. 1, with an overlapping spatial and temporal pattern [8].

In terms of malformation, for example, *Wnt1* ^*−/−*^ embryos lack the entire midbrain and cerebellum, which originates from the anterior metencephalon [24, 25]; moreover, it has been shown that inactivation of β-catenin gene by Wnt1-Cre-mediated deletion results in dramatic brain malformation and failure of craniofacial development [26].

## Brain abnormalities in CdLS

Microcephaly has been frequently reported in patients affected by CdLS, also associated with brachycephaly or plagiocephaly, both in infants and adults. No large cohort studies are available regarding type and prevalence of central nervous system anomalies in infants with CdLS (see for example Kline et al. 1993 and Selicorni et al. 2007 [27, 28]) and even fewer data are available in fetuses (see for example Avagliano et al. 2017 [29]); hence most information is related to single case reports. Parenchymal volume loss may affect both white and gray matter, especially involving midline: frontal or temporal lobes hypoplasia have been reported in possible association with hypoplasia of corpus callosum, pituitary, ventral pons and cerebellar vermian structures [30–39].

The frequency of the observed brain abnormalities in the reported cases are summarized in Table 1.

**Table 1:** Frequency of brain disorders in reported cases with deep brain investigations

Reference	Method	Microcephaly/Microencephaly	Cerebral or lobar atrophy	Brain stem size reduction	Malformation cortical development	Corpus callosum anomalies	Ventricles dilatation	Cerebellar anomalies	Cisterna magna enlargement	Pituitary
Roshan Lal 2016	MRI	4/15	6/15	3/15	1/15	3/15	3/15	5/15	5/15	4/15
Whitehead 2015	MRI and/or CT	6/7	6/8	5/7	5/8	NME	NME	6/8	NME	0/8
Lalatta 2007	MRI	1/2	1/2	0/2	0/2	1/2	0/2	1/2	0/2	0/2
Vuilleumier 2002	Autopsy	1/1	1/1	0/1	1/1	NME	0/1	0/1	0/1	0/1
Yamaguchi 1999	Autopsy	1/1	1/1	1/1	1/1	NME	NME	1/1	NME	NME
Sasaki 1996	Autopsy	1/1	1/1	1/1	1/1	NME	1/1	NME	NME	NME
Hayashi 1996	Autopsy	NME	NME	1/1	1/1	1/1	1/1	1/1	1/1	1/1
Sato 1986	Autopsy	1/1	1/1	0/1	1/1	^a^	0/1^a^	0/1	NME	^a^
France 1969	Autopsy	2/2	NME	2/2	2/2^c^	NME	1/2	1/2	NME	1/2
Mc Artur 1967	Autopsy	1/1	1/1	NME	0/1	NME	NME	0/1	NME	0/1
Hart 1965	Autopsy	1/1	NME	1/1	1/1	NME	NME	NME	NME	1/1
Schlesinger 1963	Autopsy	2/2	2/2	NME	2/2	NME	NME	NME	NME	2/2^b^

Data are presented as “number of affected cases/number of total investigated cases”

CT = computed tomography, MRI = magnetic resonance imaging, NME = no explicitly mentioned

^a^Involved by tumor invasion

^b^Histological changes

^c^Not severe gyral abnormalities but histological signs of malformation of cortical development

**Fig. 2:**
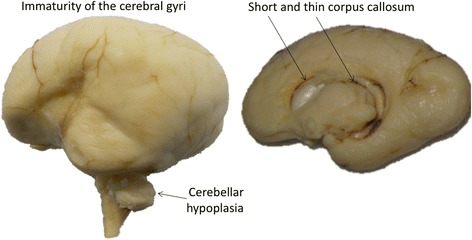
Example of brain abnormalities in a fetus at 25 weeks of gestation. Note the size reduction of the cerebellum with severe volume loss; the immaturity of sulcal pattern with slight underdevelopment of the frontal operculae for the gestational age, resulting in uncovering of the insula; the abnormalities of the corpus callosum that appears thin (in the trunk area) and short (with absence of the genu)

Corpus callosum abnormalities have been resolved by magnetic resonance imaging (MRI) and described as short or thin or hypoplastic [37, 39] (Fig. 2). Such abnormalities have also been reported at autopsy showing thin corpus callosum with rudimentary septum pellucidum in a case of CdLS associated with septo-optic dysplasia [40].

Mild dilatation of the ventricles system could be appreciated by MRI [39, 41, 42] or noted by neuropathological investigation [43] with observation of moderated dilatation of third [43] or fourth ventricle [33, 40], cerebral aqueduct and central canal of the spine [40].

Pituitary abnormalities have been reported including: reduction in size of the gland [39, 40, 44], cysts [39, 43, 45] and tumors such as microadenoma [39] or one case of invasion of the hypophysis by suprasellar germinoma [46]. In this last case the hypophysis was large. Other reports showed an unremarkable pituitary gland [31, 36–38]. Histologically, few cases were reported showing absence of basophilic cells [30, 40, 43].

Brain stem abnormalities have been observed radiologically with volume loss predominantly involving the pons [38, 39]. Autopsy data also report cases of reduced brain stem [33, 35, 43] with a specific reduction of the ventral portion of the pons [33, 35].

Cerebellum showed volume loss at head computed tomography imaging [38] and MRI [38], possibly revealing vermian hypolplasia [37, 39, 42]. Autopsy reports showed size reduction of the cerebellum (Fig. 2) [35, 43] and partial aplasia of the vermis involving the posterior part [40]. Histologically, cases with a focal loss of Purkinje cells [40] and granular cells [35, 40] have been reported associated with the presence of rudimentary superior cerebellar peduncles [40]. Heterotopic cell nests have been sometimes reported in the cerebellar white matter [35] whereas in other cases normal neuronal density and morphology have been described [36].

Cisterna magna has been described radiologically [39, 42] and anatomically [40] as enlarged up to “mega” cisterna magna.

Localized or diffuse malformations of cortical development have been frequently reported by neuroimaging [38], described as abnormal convolution characterized by gyral simplification [38, 39]. Autopsy findings also showed abnormal sulcal pattern [44] characterized by immaturity of the cerebral gyri (Fig. 2) [32, 35] with simple convolution pattern [32, 35], few shallow sulci running randomly [40] or abnormally oriented gyri resulting in strongly vertical pitch [36]. Other reports showed unduly narrow cerebral gyri [30, 33] with marked kinking [33] and wide intergyral sulci [30]. Other cases reported a relatively normal pattern of convolution with slight underdevelopment of the frontal operculae resulting in partial uncovering of the insula (Fig. 2) [43]. Histologically, focal zones of ectopic neurons have been reported, scattered in the subcortical white matter [40], as well as neuronal loss [40], and reduction in the number of oligodendroglia [33] with impaired myelinization [43, 47]. It has been suggested that the observation of focal demyelinization in CdLS may be related to incomplete myelin formation [36] rather than myelin degeneration as previously proposed [30]. Sometimes gliosis has been reported [30, 39, 40, 43] whereas in other cases was absent [32, 33, 36] suggesting that neurodegenerative changes are not specific features [36].

**Fig. 3:**
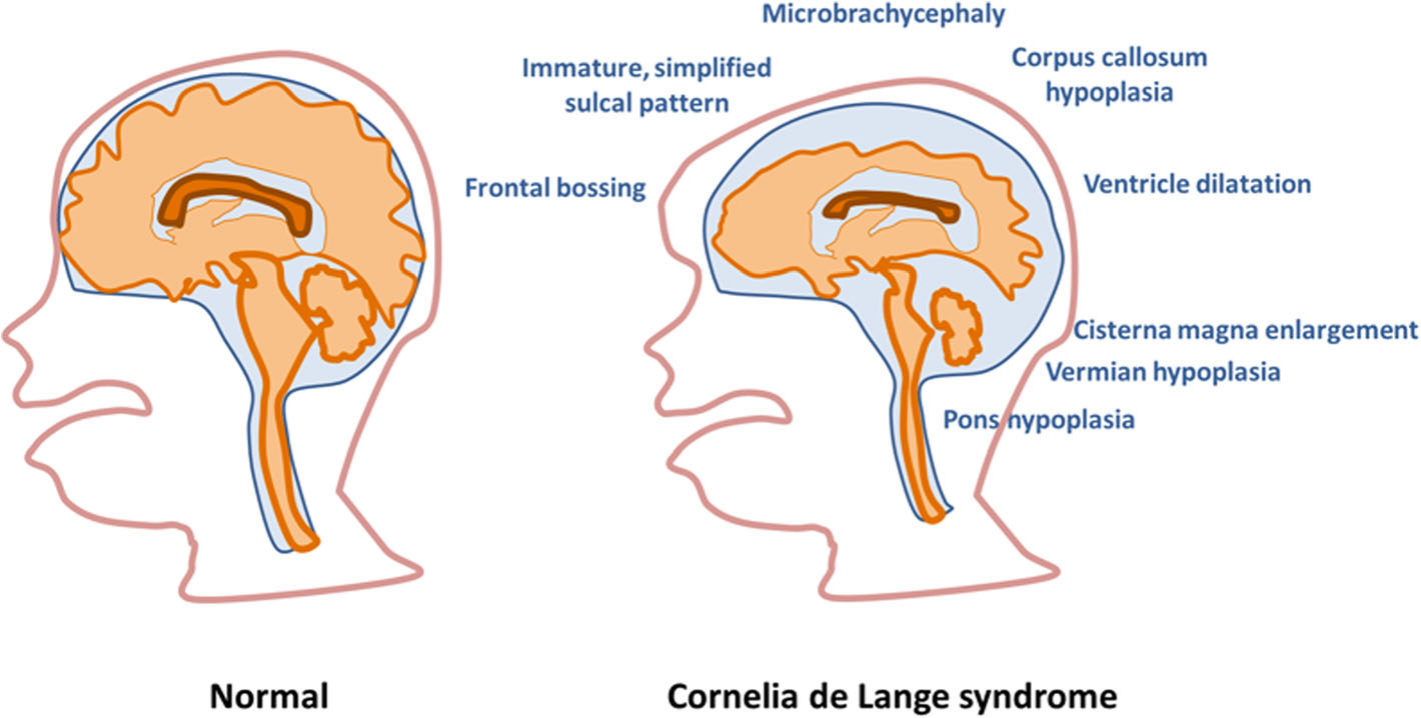
Summary of the brain abnormalities in CdLS. Cartoons compare the sagittal section of normal brain (on the left) with the sagittal section of affected brain (on the right). In the same patient, often more than one of the presented brain abnormalities are present, however only seldom all lesions are detected in the same patient

Brain abnormalities in CdLS are summarized in Fig. 3.

## Wnt signaling pathway in Cornelia de Lange

Several experimental models have been used to dissect molecular mechanisms underlying CdLS. In particular, gene expression alterations have been assessed using *D. melanogaster* [48, 49], *D. rerio* [50–54], mutant mouse [1, 2, 55] and human cells [5, 51, 54].

**Fig. 4:**
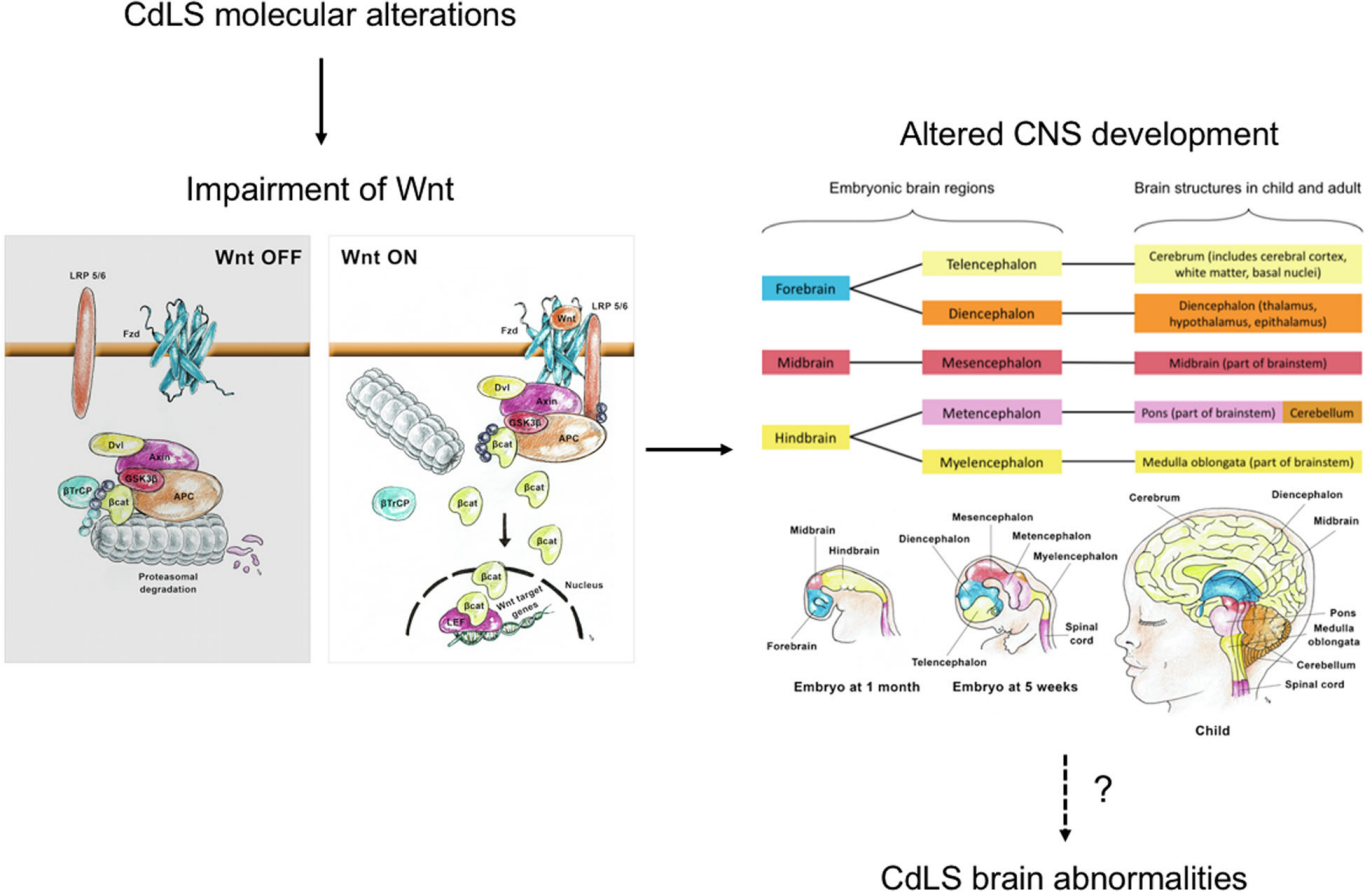
Diagram of link between CdLS, Wnt pathway and brain development. Possible link between molecular alterations and brain abnormalities in CdLS. An impairment in Wnt pathway leads to abnormalities in brain development during development that could represent one of the causes of CdLS brain malformations

In all studied models, abnormalities in the Wnt signalling pathway have been reported: gene-expression alterations in Wnt components have been shown in fibroblasts derived from patients [5, 51, 54]. In Drosophila, Nipped-B, ortholog of mammalian NIPBL, is known to regulate Ultrabithorax [56] a key repressor in the dorsoventral patterning of Wingless [57], mammalian ortholog of Wnt. Moreover, in a recent paper investigating *Rad21* reduced levels in zebrafish embryos, it was shown that heart looping is altered due to failure of neural crest cells to populate developing heart. Analysis of trascriptome revealed that among others, Wnt signalling pathway components were mis-expressed in *rad21*-depleted embryos [58]. In *D. rerio* and human fibroblasts modeling *Nipbl* [51] and *Smc1a* [54] haploinsufficiency, alterations of protein levels of the canonical Wnt pathway components have been reported. Intriguingly, in these models, chemical activation of canonical Wnt pathway has been shown to rescue the adverse phenotypes in the developing nervous system, suggesting a causative role of Wnt pathway alterations in brain abnormalities in CdLS (Fig. 4).

## Conclusions

In conclusion, this rare but severely debilitating syndrome, although described in the 1930s, is still in search of a mechanistic and neurological definition. The syndrome presents an array of congenital malformations but it is invariably associated with cognitive impairment that somehow overlaps with the so-called “autism spectrum disorders”. Here, we summarize findings that link a molecular pathway found to be perturbed in all studied models with brain morphological developmental abnormalities reported in CdLS patients, summarizing molecular and structural features that could be responsible for, at least in part, the cognitive alterations. Importantly, given the genetic heterogeneity it will be important to dissect possible differences between patients in future studies.

## Abbreviations

CdLS: Cornelia de Lange Syndrome
CT: Computed tomography
MRI: Magnetic resonance imaging
